# Insight Into the Anti-staphylococcal Activity of JBC 1847 at Sub-Inhibitory Concentration

**DOI:** 10.3389/fmicb.2021.786173

**Published:** 2022-01-05

**Authors:** Troels Ronco, Line H. Kappel, Maria F. Aragao, Niccolo Biagi, Søren Svenningsen, Jørn B. Christensen, Anders Permin, Lasse Saaby, Kim Holmstrøm, Janne K. Klitgaard, Artur J. Sabat, Viktoria Akkerboom, Monica Monaco, Marco Tinelli, Alexander W. Friedrich, Bimal Jana, Rikke H. Olsen

**Affiliations:** ^1^Department of Veterinary and Animal Sciences, Faculty of Health and Medical Sciences, University of Copenhagen, Copenhagen, Denmark; ^2^Research Unit of Molecular Microbiology, Department of Biochemistry and Molecular Biology, University of Southern Denmark, Odense, Denmark; ^3^Department of Chemistry, Faculty of Science, University of Copenhagen, Copenhagen, Denmark; ^4^Unibrains, Virum, Denmark; ^5^Bioneer A/S, Hørsholm, Denmark; ^6^Research Unit of Clinical Microbiology, Institute of Clinical Research, University of Southern Denmark, Odense, Denmark; ^7^Department of Medical Microbiology, University Medical Center Groningen, University of Groningen, Groningen, Netherlands; ^8^Department of Infectious Diseases, Istituto Superiore di Sanità, Rome, Italy; ^9^Division of Infectious and Tropical Diseases, Hospital of Lodi, Lodi, Italy; ^10^Department of Biology, Boston College, Chestnut Hill, MA, United States

**Keywords:** phenothiazine derivative, antimicrobial resistance, mode of action, RNA transcriptomics, Gram-positive bacteria

## Abstract

Multidrug-resistant pathogens constitute a serious global issue and, therefore, novel antimicrobials with new modes of action are urgently needed. Here, we investigated the effect of a phenothiazine derivative (JBC 1847) with high antimicrobial activity on *Staphylococcus aureus*, using a wide range of *in vitro* assays, flow cytometry, and RNA transcriptomics. The flow cytometry results showed that JBC 1847 rapidly caused depolarization of the cell membrane, while the macromolecule synthesis inhibition assay showed that the synthesis rates of DNA, RNA, cell wall, and proteins, respectively, were strongly decreased. Transcriptome analysis of *S. aureus* exposed to sub-inhibitory concentrations of JBC 1847 identified a total of 78 downregulated genes, whereas not a single gene was found to be significantly upregulated. Most importantly, there was downregulation of genes involved in adenosintrifosfat (ATP)-dependent pathways, including histidine biosynthesis, which is likely to correlate with the observed lower level of intracellular ATP in JBC 1847–treated cells. Furthermore, we showed that JBC 1847 is bactericidal against both exponentially growing cells and cells in a stationary growth phase. In conclusion, our results showed that the antimicrobial properties of JBC 1847 were primarily caused by depolarization of the cell membrane resulting in dissipation of the proton motive force (PMF), whereby many essential bacterial processes are affected. JBC 1847 resulted in lowered intracellular levels of ATP followed by decreased macromolecule synthesis rate and downregulation of genes essential for the amino acid metabolism in *S. aureus*. Bacterial compensatory mechanisms for this proposed multi-target activity of JBC 1847 seem to be limited based on the observed very low frequency of resistance toward the compound.

## Introduction

Infections caused by multidrug-resistant pathogens constitute a worrisome one-health issue and, consequently, there is a need for novel clinical treatment solutions (Price et al., 2012; Cassini et al., 2019; Singer et al., 2019). The pipeline for novel antimicrobial seems to be drying out, despite an urgent need (Kmietowicz, 2017; Singer et al., 2019). As an alternative to a novel antimicrobial, there has been focus on identifying compounds to “re-sensitize” antimicrobial resistant bacteria to current available antibiotics. Compounds belonging to the group of phenothiazines have received particular attention for the properties to act synergistically with oxacillin against methicillin-resistant *Staphylococcus aureus* (MRSA) (Klitgaard et al., 2008), although the antimicrobial activity of phenothiazines as single-acting antimicrobials is limited, with minimal inhibitory concentration (MIC) values between 16 and 512 mg/L, depending on the specific phenothiazine and strains under evaluation (Kristiansen et al., 2007; Nehme et al., 2018). Unfortunately, the toxicity of phenothiazines has limited their clinical relevance, e.g., because they readily pass the blood–brain barrier (BBB) causing behavioral changes (Stenger et al., 2017). To hinder the latter, we have recently synthesized a phenothiazine (promazine) derivative, JBC 1847, that does not tend to pass the BBB. This derivative is a positively charged cationic amphiphilic compound containing a quaternary ammonium ion with a monoterpene side chain, which exhibits significantly improved antimicrobial activity compared with its “mother” compound (Ronco et al., 2020). It does not, however, show synergy with beta-lactams or other antimicrobials, which has led to the hypothesis that the antimicrobial mode of action of JBC 1847 is different from original phenothiazines (Ronco et al., 2020).

Previously, we have shown that JBC 1847 is superior to fusidic acid (2% topical crème) in reducing the bacterial load of MRSA in a murine MRSA wound model (Ronco et al., 2020), displays low eukaryotic cytotoxicity (Ronco et al., 2021a), and that the compound is active against Gram-positive bacteria embedded in biofilm (Ronco et al., 2021b). The latter is particularly important as biofilms constitute a major clinical challenge due to lack of penetrance of most antibiotics through the viscous biofilm matrix (Sharma et al., 2019) and/or the lack of targeting bacteria in a more slowly growing state as often observed in biofilm (Anderl et al., 2003). The aim of the present study was therefore to investigate the effect of JBC 1847 on *S. aureus* and to determine the frequency of resistance development in *S. aureus* and *Enterococcus faecium*.

## Materials and Methods

### Bacterial Strain and Antimicrobial Compounds

*Staphylococcus aureus* JE2, a plasmid-cured derivative of a MRSA isolate USA300 (Fey et al., 2013), was used as reference strain to study the effect of JBC 1847 on *S. aureus*. The isolate was stored in brain–heart infusion (BHI) broth with 15% glycerol at −80°C until used.

For determination of the frequency of resistance, two additional isolates were included: *S. aureus* CC398 and *E. faecium* ATCC 700221. As JBC 1847 and Ca^++^-activated daptomycin (DAP) are both positively charged (which likely contributes to the antimicrobial activity of the latter), it was investigated if JBC 1847 displays cross-resistance with DAP. For this purpose, a DAP sensitive wild-type strain (IT1-S) and its DAP-resistant variant (IT4-R) (Sabat et al., 2018) were applied.

The antimicrobial compounds JBC 1847 and T5 (molecular structures are presented in Supplementary Figure 1) were synthesized at University of Copenhagen, Denmark, as previously reported (Jørgensen et al., 2020; Ronco et al., 2020). All other antimicrobials were purchased at Sigma-Aldrich (Brøndby, Denmark), unless mentioned otherwise.

### Determination of the Antimicrobial Activity

The broth dilution method based on the guidelines from Clinical and Laboratory Standards Institute was applied for the determination of the MIC and the minimal bactericidal concentration (MBC) values. MIC values were determined in 96-well plates as previously described by Ronco et al. (2020). To determine MBC, 20 μl from each well in the 96-well plate from the MIC determination were inoculated on blood agar (BA) plates (blood agar base, Oxoid, Roskilde, Denmark; supplemented with 5% bovine blood), incubated at 37°C for 18–24 h and colonies were then enumerated. MBC was determined as the concentration reducing the initial bacterial concentration per well with ≥99.9%. MBC determination for cells in stationary phase was done as follows: After 24-h incubation of *S. aureus* JE2 at 37°C in Müller Hinton broth (Oxoid), the MH broth was diluted 1:4 in 0.9% saline (final volume 5 ml) and mixed with JBC 1847 to yield concentrations between 0.25 and 8 mg/L of JBC 1847. A negative control (broth without JBC 1847) was also included. All tubes were incubated at 37°C for 24 h, and the CFU per milliliter of each tube was enumerated. The MBC was determined as the lowest concentration yielding a 99.9% reduction of the CFU in the negative control. The experiment was repeated twice.

Determination of the JBC 1847 MIC in a DAP-sensitive strain of *S. aureus* and a DAP-resistant strain, respectively, were done according to Sabat et al. (2018).

### Determination of Single-Step Resistance Generation Mutations

The frequencies of single-step spontaneous mutations in five bacterial strains were determined. Log-phase bacteria at a concentration of 10^6^–10^10^ CFU/ml were plated onto BHI agar plates containing antimicrobial compounds in a concentration of 8 × MIC for each compound, and the plates were incubated at 37°C for 48 h. In addition, several dilutions of each culture were plated on drug-free media to provide accurate colony counts. Resistance frequencies were calculated by dividing the number of colonies growing on antibiotic plates by the total number of CFU plated as outlined by Butler et al. (2002). Each experiment was performed in triplicate on separate days, and mutation frequencies represent average values. To verify the stability of each resistant organism, the organisms were transferred several times in drug-free medium and medium containing a drug concentration of 4 × MIC and were again tested for resistance.

### Assessment of Membrane Potential Using Flow Cytometry

Measurement of the membrane potential using the BacLight Bacterial Membrane Potential Kit (Invitrogen) in three biological replicates was done according to Wassmann et al. (2018). Briefly, an overnight culture of *S. aureus* JE2 was diluted to an OD_600_ 0.02 in BHI and incubated at 37°C until early exponential phase (OD_600_ 0.3). The culture was then diluted 1:100 in sterile phosphate-buffered saline and split into separate tubes containing 1 ml diluted culture each. One sample was treated with 10 μM of CCCP (carbonyl cyanide 3-chlorophenylhydrazone) to depolarize the cells, thereby acting as a depolarized control; one sample was treated with DMSO, while four samples were treated with four different concentrations of JBC 1847 (0.0625–0.50 mg/L). All samples were stained for 15–30 min with 10 μM of DiOC2 dye and the last sample was left as an unstained control. After staining, the samples were analyzed in a BD FACS Aria II flow cytometer (Becton, Dickinson and Company, Franklin Lakes, NJ, United States). Hereafter, 10^4^ events were analyzed, while exciting the particles with a laser (488 nm) and collecting the green and red fluorescence.

### Isolation of Total Cellular RNA

Here, the RNA expression response of *S. aureus* JE2 to sub-inhibitory concentrations of JBC 1847 was investigated relative to the untreated control. For comparative reasons, the responses of *S. aureus* JE2 to sub-inhibitory concentrations of promazine, the “mother” compound of JBC 1847 and to T5, and another phenothiazine derivative with structural similarity to JBC1847 (Jørgensen et al., 2020), were also included in the analysis. Initially, growth curves of *S. aureus* JE2 were done to ensure that exposure of the strain to 1/4 × MIC of either JBC 1847 (0.25 mg/L), T5 (0.5 mg/L), or promazine (8 mg/L) did not impair growth compared with the untreated control (Supplementary Figure 2).

An overnight culture of *S. aureus* JE2 grown on BA was prepared and a single colony was picked and grown in MH broth for 18–20 h. The overnight culture was diluted in BHI to OD_600_ 0.02 and grown at 37°C with shaking, divided into four and treated with either 1/4 × MIC of JBC 1847, 1/4 × MIC of T5, 1/4 × MIC of promazine, or left untreated for 30 min at 37°C. The procedure was repeated three times, generating biological triplicates of the four treatment conditions. Total RNA from each sample was extracted applying the RNAeasy kit (Qiagen, Hilden, Germany) according to the manufacturer’s instruction. The samples were stored at −80°C until preparation for RNA sequencing.

### Library Preparation for RNA HiSeq Sequencing

RNA concentrations of each of the 12 samples were measured using the Qubit BR RNA assay, and the RNA quality was evaluated using TapeStation with the RNA ScreenTape (Agilent Technologies, Santa Clara, CA, United States). All samples were rRNA depleted using the Ribo-Zero Plus rRNA Depletion Kit (Illumina, Inc., San Diego, CA, United States), and residual DNA from RNA extraction was removed using the DNase MAX kit (MoBio Laboratories Inc., Carlsbad, CA, United States). The samples were purified using the standard protocol for CleanPCR SPRI beads (CleanNA, Zuid, Netherlands) and further prepared for sequencing using the NEBNext Ultra II Directional RNA library preparation kit (New England Biolabs, MA, United States). Library concentrations were measured using Qubit HS DNA assay and library DNA size estimated using TapeStation with D1000 ScreenTape. The samples were pooled in equimolar concentrations and paired-end sequenced (2 × 150 bp) using a HiSEQ X platform (Illumina, United States). All kits were used as per the manufacturer’s instructions with minor modifications.

### Transcriptome Mapping

Forward and reverse DNA reads from the raw fastq files were filtered for PhiX using Bowtie2 by aligning the reads against a database composed of the Coliphage phi-X174 genome (RefSeq accession NC_001422.1) indexed also using Bowtie2 (Langmead and Salzberg, 2012). Reads passing the PhiX-filtering were then trimmed for sequencing adaptors, quality filtered (Q-score > 20), and sequences shorter than 100 nucleotides discarded using Cutadapt (v2.10) (Marcel, 2013). Also using Cutadapt, sequencing adaptors were then trimmed from the reads passing the PhiX-filtering, and low-quality sequences (Q-score ≥ 15) and those shorter than 100 nucleotides were discarded. A rRNA reference database was constructed by concatenating the small subunit (SSU) and large subunit (LSU) rRNA SILVA Parc databases and indexing using Bowtie2. Finally, reads were bioinformatically depleted for rRNA sequences by aligning reads against the constructed SSU/LSU SILVA Parc database using Bowtie2. The genome sequence of *S. aureus* JE2 with annotations (RefSeq accession GCF_002085525.1) was further annotated using Prokka (v1.14.6) (Seemann, 2014), and the resulting genome was indexed using Bowtie2. The rRNA-depleted and quality-filtered DNA reads were aligned against the genome with the Bowtie2–very-sensitive option, and all alignments were ported to unsorted .sam files. The .sam files from Bowtie2 were then sorted by sequence gene identifier (prokkaID) and converted to .bam files using samtools (v1.10). Finally, transcript/gene count tables were made using featureCounts (v2.0.1) (Li et al., 2009). Where nothing else is stated, the default settings were used for all tools. The DESeq2 workflow was used to normalize read counts by the geometric mean count and identify differentially expressed genes. Importantly, rRNA gene expression levels were not included as both the RNA sample preparation and subsequent bioinformatic processing included rRNA depletion. Samples treated with either T5, JBC1847, or promazine were compared against the experiment control (Ctrl), and changes in gene transcript counts were considered statistically significant with the dual criteria of the observed fold change greater than twofold and the Benjamini–Hochberg corrected *p*-value < 0.05.

### Differential Gene Expression Analysis

The transcript/gene count tables were imported to RStudio (R Core Team, 2021) and processed using the default DESeq2 workflow and visualized using ggplot2. Principal component analysis (PCA) of overall sample similarity was done using the log-transformed, DESeq2 normalized counts.

### Metabolic Modulation

For the functional annotation of the transcriptomes, the protein FASTA file of the translated CDS sequences (PROKKA_03112021.faa) was processed using the kyoto encyclopedia of genes and genomes (KEGG) Automatic Annotation Server Ver. 2.1 (KAAS, last update April 3, 2015) for automatic annotation and pathway reconstruction (Moriya et al., 2007). The bi-directional best hit (BBH) method was used to assign orthologs to sequences and the representative gene data set was set to prokaryotes. All other settings were left to default. The KEGG BRITE database (hierarchy file text), relating KO (KEGG Orthology) numbers with pathway information, was downloaded from https://www.kegg.jp/kegg-bin/get_htext?ko00001.keg (June 22, 2021) (Kanehisa and Goto, 2000; Kanehisa, 2019; Kanehisa et al., 2021). Files were cleaned retaining only the relationship between KO number and hierarchy level B. Barplots displaying the distribution of up- and downregulated genes per pathway were constructed using the predicted KO orthologs, the cleaned KEGG BRITE database, and identified differential expressed genes (DEGs) between conditions. All bioinformatic processing was done *via* RStudio IDE (1.2.1335) running R version 4.0.3 (October 10, 2020) (R Core Team, 2021) and using the R packages tidyverse (1.3.1) and ggplot2 (3.3.5).

### Macromolecule Biosynthesis

To determine the possible impact of JBC 1847 on the synthesis rate of DNA, RNA, cell wall, and proteins, respectively, a macromolecule biosynthesis assay was conducted essentially according to Jana et al. (2017) with a modified protocol adapted to 96-well plates. Briefly, an overnight *S. aureus* JE2 culture was diluted 1:100 in Mueller Hinton Broth 2 (Millipore) and sub-cultured to OD_600_ of 0.2. One milliliter of culture was distributed into each of four 1.5-ml Eppendorf tubes and the four radiolabeled precursors [(3H)-thymidine (Perkin Elmer), (3H)-uridine (Perkin Elmer), (3H)-isoleucine (American Radiolabeled Chemicals Inc.), and (3H)-glucosamine hydrochloride (American Radiolabeled Chemicals Inc.)], respectively, were added to a concentration of 0.1 mCi/ml. For each isotope, after vortexing, 100-μl volumes were immediately transferred to four microtiter wells to create two sets of samples with a technical replicate (two for JBC1847 and two for solvent control). To one pair of wells a concentration corresponding to 0.75 × MIC of JBC 1847 was added and to a second pair of wells solvent was added corresponding to the concentration of solvent in the JBC 1847 samples. All wells were incubated/pulse labeled for 20 min at 37°C. Subsequently, radiolabeled cells were precipitated with equal volume (110 μl) of ice-cold 30% trichloroacetic acid (TCA) and left on ice for 1–2 h. Cell precipitates (220 μl) were transferred to 96-well format cellulose membrane filters (UniFilter GF/C, Perkin Elmer) using a FilterMate Harvester (Perkin Elmer), and were subsequently washed two times with 200 μl ice-cold 15% TCA and one time with 200 μl ice-cold water. The filter plate was dried overnight before 20 μl of scintillation fluid was added to each well and 3H counts were obtained using a MicroBeta2 Microplate Reader (Perkin Elmer). Macromolecule biosynthesis rates were calculated based on the assumption that the rates of DNA, RNA, cell wall, and protein precursor incorporation were 100% in the control sample (treated with JBC 1847 solvent solution).

### Intracellular Adenosintrifosfat and Adenosine Diphosphate Measurements

To investigate whether JBC 1847 affects the intracellular concentration of adenosintrifosfat (ATP) in *S. aureus* JE2 and/or the ATP/adenosine diphosphate (ADP) ratio, an ATP/ADP measurement assay was carried out. To measure intracellular ATP/ADP concentrations, JBC 1847 was initially dissolved in PBS to 40 mg/L, which was further diluted to 2.5 and 10 mg/L solutions in PBS. *S. aureus* JE2 was inoculated in Mueller Hinton Broth II (MHB II) growth media and cultured overnight at 37°C with agitation (150 rpm). The overnight culture was sub-cultured at 1:1,000 dilution in 25 ml MHB II media and incubated at 37°C including agitation (150 rpm), until reaching an OD_600_ 0.1. The resulting OD_600_ 0.1 culture was portioned in aliquots of 9 ml in suitable vials and incubated for 4 h. Subsequently, the prepared JBC 1847 solutions were added in appropriate volumes to yield final concentrations of mg/L JBC 1847. *S. aureus* incubated with blank PBS served as a negative control. Each treatment (4 mg/L JBC 1847 or untreated negative control) was tested in triplicate (*n* = 3). The resulting mixtures were incubated (37°C, 150 rpm). Samples of 1 ml were harvested after *t* = 15 and *t* = 60 min of incubation. Samples were immediately centrifuged at 13,000 rpm for 10 min and the resulting pellet was re-suspended in MilliQ water. The resulting cell suspension was lysed by bead-beating and centrifuged at 13,000 rpm for 10 min. The supernatant was analyzed using the ADP/ATP kit (ADP/ATP Ratio assay kit; Sigma-Aldrich) according to the manufacturer’s instruction.

### Lysis of Erythrocytes

The degree of compound-induced lysis of erythrocytes was investigated at concentrations up to 25 mg/L of JBC 1847. Thus, JBC 1847 was dissolved in PBS to an initial concentration of 25 mg/L and treated with ultrasound. The initial stock solution was further diluted in PBS to produce solutions with concentrations of 1 and 5 mg/L and 300 μl bovine whole blood was then mixed with 1,200 μl sample solution. The mixture of whole blood and sample solution was agitated for 30 min by end-over-end rotation at ambient temperature. Subsequently, the mixture was centrifuged for 10 min at 13,000 rpm. The supernatant was diluted 20-fold in ultrapure water and the absorbance of the diluted supernatant was measured at 540 nm (LabSystems Multiscanner plate reader). All samples were measured in duplicates. PBS and isotonic saline were included as negative controls where no lysis of red blood cells was expected. Ultrapure water and a solution of 0.1% Triton X-100 (AppliChem GmbH, Darmstadt, Germany) in PBS were included as positive controls where complete lysis of erythrocytes was expected.

## Results

### Minimal Inhibitory Concentration, Minimal Bactericidal Concentration, and JBC 1847 Resistance Development

The MIC of JBC 1847 was determined to equal 1 mg/L for both *S. aureus* JE2 and *S. aureus* CC398, and 2 mg/L for *E. faecium*. In all cases, culturing of 20 μl from the wells corresponding to the MIC values revealed a CFU/ml reduction of ≥99.9% compared with the initial inoculum in the wells. Thus, the MIC of JBC 1847 for these strains equaled the MBC. The MBC of JBC 1847 was equal to the MBC of exponential growing cells.

The MIC for both the DAP-sensitive IT1-S strain and the DAP-resistant variant equaled 1.5 mg/L, indicating that DAP resistance does not cause cross-resistance to JBC 1847.

When investigating frequencies of single-step spontaneous mutations at plates containing either 4 × MIC (4 mg/L) or 8 × MIC (8 mg/L) of JBC 1847, not a single *S. aureus* or *E. faecium* mutant could be found. Hence, the resistance frequency of single-step resistance-generating mutation(s) was <7.33 × 10^–7^, whereas the resistance frequencies for mupirocin and fusidic acid varied between 1.28 × 10^–4^ and 2.82 × 10^–8^ (Table 1), depending on the strain, antimicrobial, and concentration.

**TABLE 1:** Frequencies of single-step spontaneous mutations.

	JBC 1847		Fusidic acid		Mupirocin	
Strain	4 × MIC	8 × MIC	4 × MIC	8 × MIC	4 × MIC	8 × MIC
MRSA USA300	<2.32 × 10^–7^*	<2.32 × 10^–7^*	1.44 × 10^–6^ (SD 8.9 × 10^–4^)	5.96 × 10^–7^ (SD 3.6 × 10^–4^)	1.28 × 10^–4^ (SD 4.2 × 10^–5^)	1.83 × 10^–5^ (SD 1.1 × 10^–5^)
MRSA CC398	<7.33 × 10^–6^*	<7.33 × 10^–6^*	1.45 × 10^–6^ (SD 3.1 × 10^–4^)	1.18 × 10^–6^ (SD 2.5 × 10^–4^)	<7.33 × 10^–6^*	<7.33 × 10^–6^*
*E. faecium*	<8.07 × 10^–7^*	<8.07 × 10^–7^*	3.08 × 10^–7^ (SD 7.3 × 10^–7^)	2.82 × 10^–8^ (SD 6.3 × 10^–7^)	1.37 × 10^–6^ (SD 1.4 × 10^–6^)	<5.01 × 10^–7^*

Standard deviations (SD) are shown for assays revealing a least one mutant. *SD could not be calculated as single mutant was not observed in any of the three assay replicates.

### JBC 1847 Dissipates the Cytoplasmic Membrane Proton Motive Force

**FIGURE 1:**
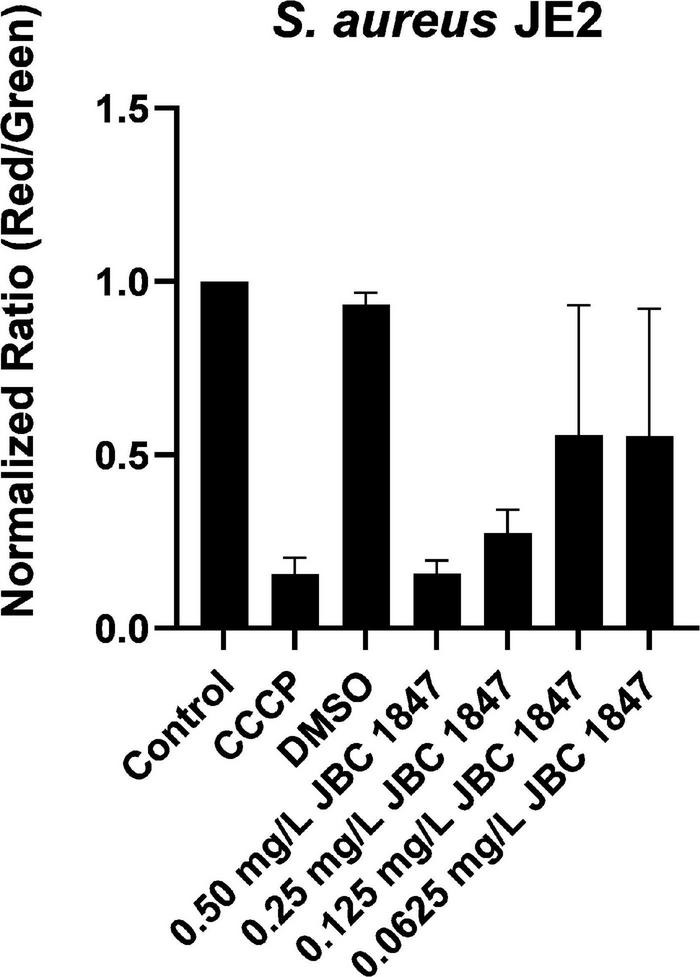
Assessment of membrane potential using flow cytometry. Membrane depolarization assay showing the effect of 0.0625–0.50 mg/L JBC 1847 on the membrane potential of *Staphylococcus aureus* JE2. Bar chart showing red/green mean fluorescence intensity ratio where a high ratio indicates high membrane potential and a low ratio a depolarized membrane. CCCP (2-[2-(3-chlorophenyl)hydrazinylyidene]propanedinitrile) is an oxidative phosphorylation uncoupler used as a positive control for membrane potential depolarization. Data are normalized against untreated control and show mean values of three biological replicates and SDs.

The depolarization effects of JBC 1847 on cell membrane potential depolarization were observed to be concentration dependent (Figure 1). CCCP was used as positive control for depolarization of the membrane. The same depolarizing effect can be observed when comparing *S. aureus* JE2 exposed to sub-inhibitory concentrations of JBC 1847 at 0.5 mg/L (1/2 MIC). These observations indicate that JBC 1847 strongly dissipates the cytoplasmatic membrane proton motive force (PMF).

### JBC 1847 Inhibits Macromolecule Biosynthesis

**FIGURE 2:**
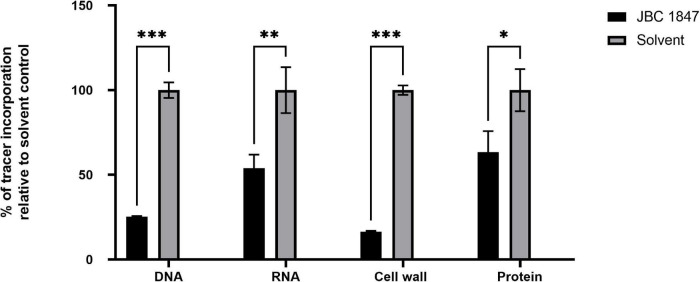
Macromolecular synthesis assay in the presence of JBC 1847 and control (JBC 1847 solvent). Incorporation of radiolabeled precursors such as [3H] thymidine, [3H] uridine, [3H] leucine, [14C] N-acetylglucosamine, and [3H] glycerol for DNA, RNA, protein, and cell wall synthesis, respectively, were quantified in *Staphylococcus aureus* JE2. Based on the incorporation of radiolabeled precursors, percent of inhibition by JBC 1847 was examined. Two biological replicates were used for each group and the statistical analysis was calculated by the two-tailed Student *t*-test. Each of the macromolecule groups were compared to the associated untreated control group. *p < 0.05; **p < 0.01; ***p < 0.001.

The synthesis rates of all tested macromolecules (DNA, RNA, cell wall, and protein) were significantly decreased in *S. aureus* JE2 exposed to JBC 1847 for 20 min (Figure 2). In particular, the DNA and cell-wall synthesis rates were highly affected by JBC 1847 exposure.

### RNA Sequencing Results

**FIGURE 3:**
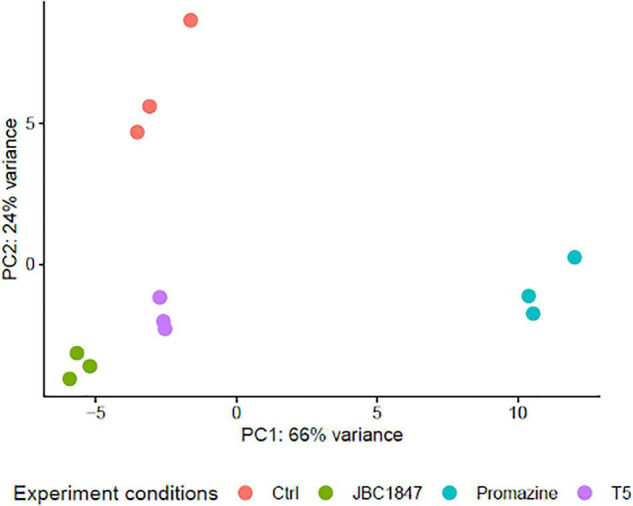
Principal component analysis (PCA) of gene expression profiles. PCA of *Staphylococcus aureus* JE2 transcriptomes when exposed to sub-inhibitory concentrations of either promazine, JBC 1847 (a promazine derivative), T5 (a thioridazine derivative), or left as untreated control (Ctrl). Closeness between sample points indicates transcriptome similarity such that the closer points are together, the more similar gene expression profiles. For axes interpretation, please note the variance contribution of each principal component.

**FIGURE 4:**
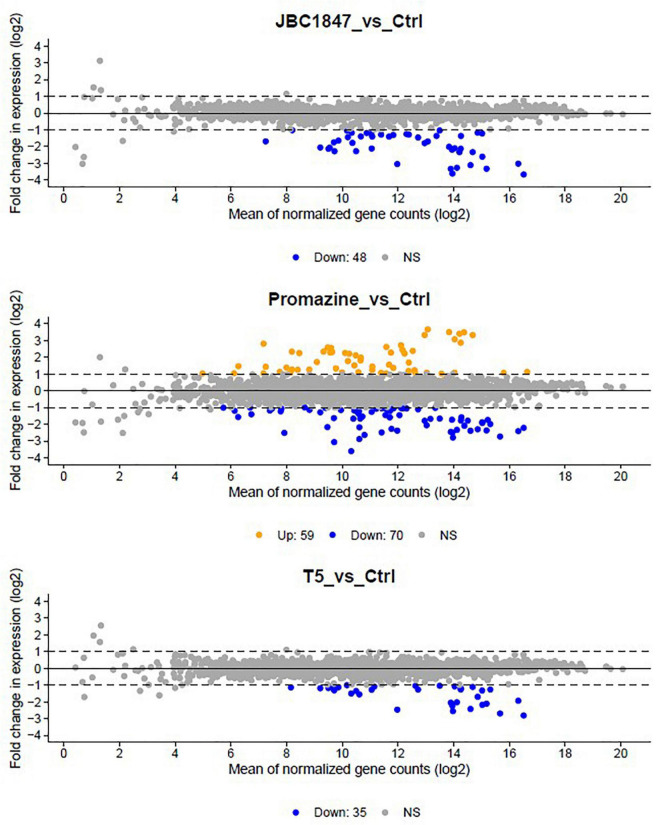
MA plots of gene expression in *Staphylococcus aureus* JE2. Visualization of gene expression of *S. aureus* exposed to JBC 1847 vs. untreated control (Ctrl) (upper figure), gene expression of *S. aureus* exposed to promazine vs. Ctrl (middle figure), and gene expression of *S. aureus* exposed to T5 vs. Ctrl (lower figure). The plot visualizes the differences between RNA levels in the two samples, by transforming the data onto M (log ratio) and A (mean average) scales, then plotting these values. Hence, the MA plots show the fold change in gene expression between experiment treatment groups vs. the untreated control (Ctrl) as a function of the averaged, normalized transcript count of each gene. Each point corresponds to one gene. Statistically significant up- and downregulated genes are shown in orange and blue, respectively. Non-significant (NS) changes are in gray. The axes are logarithmic (log2) and dashed lines indicate the *y*-axis levels corresponding to a twofold change in gene expression. Notice the complete lack of orange points in the upper and lower plots, indicating that none of the genes in *S. aureus* were upregulated by treatment with JBC 1847 or T5. In contrast, several genes were either up- or downregulated in promazine-treated *S. aureus*.

To investigate how JBC 1847 influences gene expression, the RNA expression profile was investigated in *S. aureus* JE2 exposed to sub-inhibitory concentrations of JBC 1847. The total number of raw DNA sequence reads (17–26 mio reads per sample) were nucleotide quality and phi-X filtered (generating between 14 and 22 mio reads per sample) before mapping to the reference genome. On average, the ratio of the number of reads mapping to the reference compared with the number of quality filtered DNA reads was 0.993. Sample-wise PCA of the DESeq2-normalized gene expression data indicated that the samples readily cluster according to the experiment treatment groups (Figure 3). Figure 4 shows that all significantly regulated genes in JBC 1847 and T5 treated *S. aureus* were downregulated. Overall, the same genes were affected by T5 as JBC 1847 supporting the close approximation in the PCA plot of JBC 1847 and T5 treatments. In contrast, promazine-treated *S. aureus* exhibited a different expression profile where genes were both up- and downregulated (Figure 4). For JBC 1847 (and T5) treatments, genes related to the histidine biosynthesis and nitrate metabolism were the most abundant groups of regulated genes (Table 2). The RNA sequence data have been deposited in the Sequence Read Archive (SRA) from NCBI under the BioProject ID PRJNA766628.

**TABLE 2:** The 30 genes with lowest *p*-values and highest 2 log-fold changes (downregulated) in *Staphylococcus aureus* JE2 exposed to 30 min of sub-inhibitory concentrations of JBC 1847 vs. untreated control conditions.

Gene	Product	Fold change	*P*-value
*narH*	Nitrate reductase subunit beta	−4.07	1.75 × 10^–148^
*narJ*	Reductase molybdenum cofactor assembly chaperone	−4.02	1.23 × 10^–165^
*narG*	Nitrate reductase subunit alpha	−3.66	4.96 × 10^–113^
*narI*	Respiratory nitrate reductase subunit gamma	−3.61	9.14 × 10^–288^
*nreA*	Respiration regulation sensor histidine kinase NreA	−3.32	2.89 × 10^–200^
*nreB*	Nitrate respiration regulation sensor histidine kinase NreB	−3.32	1.10 × 10^–218^
*nreC*	Nitrate respiration regulation response regulator NreC	−3.26	1.21 × 10^–259^
*cobA*	Uroporphyrinogen-III C-methyltransferase	−3.10	1.64 × 10^–96^
*nirD*	Nitrite reductase small subunit NirD	−3.04	6.0 × 10^–81^
*narK*	NarK/NasA family nitrate transporter	−3.02	3.10 × 10^–174^
*nasD*	NAD(P)/FAD-dependent oxidoreductase	−2.60	2.91 × 10^–138^
*dapB*	4-Hydroxy-tetrahydrodipicolinate reductase	−2.33	1.92 × 10^–45^
*dapA*	4-Hydroxy-tetrahydrodipicolinate synthase	−2.33	2.57 × 10^–19^
*hisB*	Imidazole glycerol-phosphate dehydratase HisB	−2.27	1.99 × 10^–36^
*hisC*	Histidinol-phosphate aminotransferase family protein	−2.27	3.24 × 10^–44^
*hisH*	Imidazole glycerol phosphate synthase subunit HisH	−2.13	1.22 × 10^–44^
*hisD*	Histidinol dehydrogenase	−2.11	1.36 × 10^–35^
*hisA*	Phosphoribosylformimino-5-aminoimidazole carboxamide ribotide isomerase	−2.06	7.15 × 10^–35^
*hisZ*	Adenosintrifosfat (ATP) phosphoribosyltransferase	−2.05	1.12 × 10^–35^
*hisE*	Bifunctional phosphoribosyl-AMP Cyclohydrolase/phosphoribosyl-ATP diphosphatase HisIE	−1.77	2.32 × 10^–38^
*hisF*	Imidazole glycerol phosphate synthase subunit HisF	−1.74	7.73 × 10^–35^
*dapD*	2,3,4,5-Tetrahydropyridine-2,6-dicarboxylate N-acetyltransferase	−2.01	2.50 × 10^–82^
*ndhF*	NADH dehydrogenase subunit 5	−1.6	2.20 × 10^–21^
*ilvC*	Ketol-acid reductoisomerase	−1.44	6.26 × 10^–25^
*ilvB*	Biosynthetic-type acetolactate synthase large subunit	−1.40	4.66 × 10^–18^
*ilvD*	Dihydroxy-acid dehydratase	−1.22	2.82 × 10^–11^
*leuA*	2-Isopropylmalate synthase	−1.39	1.26 × 10^–21^
*YbcC*	YbcC family protein	−1.37	3.70 × 10^–26^
*argH*	Argininosuccinate lyase	−1.36	3.39 × 10^–22^
*argG*	Argininosuccinate synthase	−1.25	6.42 × 10^–40^
*escA*	ATP-binding cassette domain-containing protein	−1.40	4.22 × 10^–12^
*asd*	Aspartate-semialdehyde dehydrogenase	−2.10	2.20 × 10^–43^
*mnh*	Na^+^/H^+^ antiporter family protein	−1.22	7.70 × 10^–31^
*rlp*	Peptide ABC transporter substrate-binding protein	−1.28	7.15 × 10^–18^
*lrgA*	Antiholin-like murein hydrolase modulator LrgA	−1.17	5.98 × 10^–6^
*cbiX*	Sirohydrochlorin chelatase	−2.18	1.82 × 10^–35^

*No significantly upregulated genes were identified in the same transcriptomic analysis.*

### Kyoto Encyclopedia of Genes and Genomes Metabolic Modeling

**FIGURE 5:**
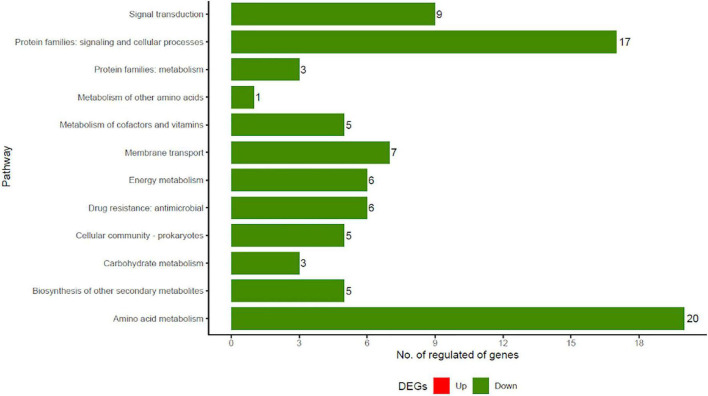
Kyoto encyclopedia of genes and genomes (KEGG) functional classification of regulated genes in *Staphylococcus aureus* JE2 exposed to JBC 1847. Only genes significantly differently expressed from genes in the untreated control are included. Note the absence of red bars (upregulated genes) among the differentially expressed genes (DEGs). The number of genes assigned to each pathway is stated to the right of the bars.

To further investigate the metabolic pathways of *S. aureus* treated with JBC 1847, a KEGG enrichment analysis of DEGs was performed. The DEGs were assigned to 12 KEGG bioprocess pathways (Figure 5), of which all were downregulated compared with the untreated control. Genes belonging to signaling and cellular processes as well as amino acid metabolism were the most abundant (Figure 5). JBC 1847 exposure downregulated genes that are involved in histidine biosynthesis, nitrate reduction, and ATP-dependent bioprocesses (Table 2).

### JBC 1847 Decreases Intracellular Adenosintrifosfat and Increases the Adenosintrifosfat/Adenosine Diphosphate Ratio

**FIGURE 6:**
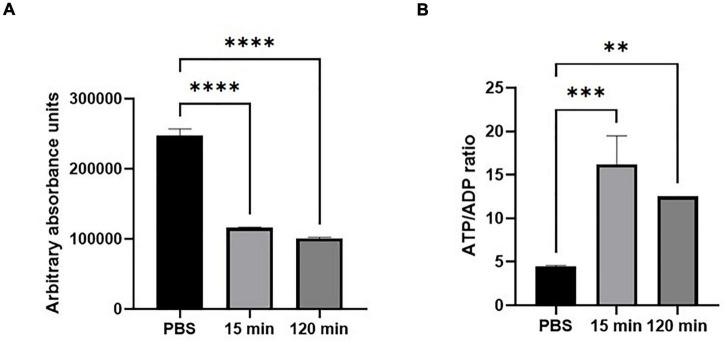
Intracellular adenosintrifosfat (ATP) level (measured in absorbance units) **(A)** and ATP/adenosine diphosphate (ADP) ratio **(B)** in *Staphylococcus aureus* after exposure to JBC 1847 (4 mg/L) in either 15 or 120 min. The error bars show SD of three replicates. **p < 0.01; ***p < 0.001; ****p < 0.0001.

To investigate if JBC 1847 influences the intracellular concentration of ATP and/or the ATP/ADP ratio in *S. aureus* JE2, an ATP/ADP measurement assay was carried out. The results showed a marked reduction of intracellular ATP levels in *S. aureus* JE2 exposed to JBC 1847 for 15 min (Figure 6A). Compared with the PBS control, the ATP level remained significantly lower, even after 2 h. In contrast, the ATP/APD ratio was significantly higher in cells exposed to JBC 1847 (Figure 6B).

### Erythrocyte Lysis

**FIGURE 7:**
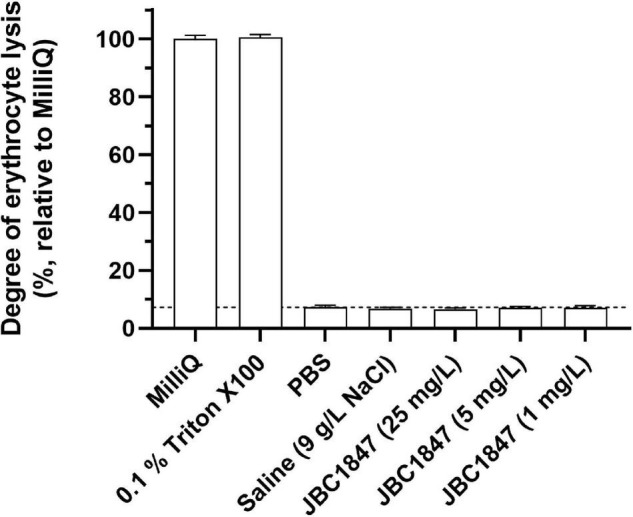
Evaluation of the hemolytic activity of JBC 1847. The assay included concentrations of JBC 1847 ranging from 1 to 25 mg/L.

Since JBC 1847 exhibits a cationic amphiphilic structure, its hemolytic activity was investigated. The lysis assay did not show any hemolytic activity of JBC 1847 evaluated in concentrations up to 25 mg/L (Figure 7), indicating JBC 1847 is at least partly selectively targeting the bacterial membrane of *S. aureus*.

## Discussion

Novel antimicrobials with new modes of action and low potential for bacterial resistance development are highly needed (Singer et al., 2019). Here, we have assessed the effect of JBC 1847, a potential novel antimicrobial, on *S. aureus*.

JBC 1847 is a cationic amphiphilic compound and has a quaternary ammonia structure and a constantly positive charge. The latter shares similarity to the very potent antimicrobial, daptomycin (DAP). DAP forms a positively charged complex with Ca^++^, which is a strict requirement for the activation of DAP (Grein et al., 2020). Both micelle formation of DAP and the positive charge of the DAP–Ca^++^ complex are assumed to contribute to the antimicrobial activity of DAP (Miller et al., 2016). However, in the present study we have shown that a DAP-resistant strain is not (cross-) resistant to JBC 1847; hence, the bacterial target(s) of JBC 1847 must be different from the target(s) of DAP. While the transcriptomic analysis revealed that a unique set of *S. aureus* genes were upregulated when exposed to sub-lethal concentrations of promazine, there was a complete lack of any upregulated genes in *S. aureus* JE2 in the presence of JBC 1847 (Figures 4A,C). Furthermore, the JBC 1847 exposed transcriptomes of *S. aureus* JE2 were strongly separated from the promazine transcriptomes in the PCA, indicating a highly different expression profile of *S. aureus* JE2, depending on treatment. Concordantly, we suggest that JBC 1847 has a different effect on *S. aureus* compared with its “mother” compound, promazine. The fact that *S. aureus* JE2 reacts differently to JBC 1847 exposure than to promazine exposure is not surprising since the MIC in *S. aureus* is considerably lower for JBC 1847 than for promazine. In this study, the MIC for JBC 1847 was found to be 1 mg/L whereas the MIC for promazine previously has been determined to be 128 mg/L in *S. aureus* JE2 (Jørgensen, 2020) and in other strains of *S. aureus* (Nehme et al., 2018). Furthermore, JBC 1847 is not acting synergistically with oxacillin whereas this property has been reported for various phenothiazines (Klitgaard et al., 2008; Hadji-Nejad et al., 2010) and previously we have shown *via in silico* analysis that JBC 1847 exhibits different pharmacological parameters than promazine (Ronco et al., 2020, 2021a). In contrast to promazine, the transcriptomic profile of *S. aureus* exposed to sub-inhibitory concentrations of JBC 1847 is highly similar to transcriptomic profile of *S. aureus* exposed to sub-inhibitory concentrations of T5, another phenothiazine [thioridazine (10-[2-(1-methylpiperidin-2-yl)ethyl]-2-methylsulfanylphenothiazine) (Wassmann et al., 2018)] derivative (Jørgensen et al., 2020). This indicates that the structurally similar, positively charged side chains added to thioridazine as well as promazine to generate T5 and JBC 1847, respectively, is of greater importance for the antimicrobial activity than which exact phenothiazine the derivatives originate from. For both T5 and JBC 1847, all DEGs were exclusively downregulated (Figure 4), with genes in pathways belonging to amino acid metabolism, nitrate reduction, and cell signaling as the most abundant DEGs (Table 2 and Figure 5). Among genes downregulated within amino acid metabolism, genes of the histidine metabolism were particularly abundant (Table 2). Downregulation of genes involved in the histidine metabolism has been shown to be crucial for cefquinome-induced biofilm inhibition of *Staphylococcus xylosus* (Zhou et al., 2018). It is therefore likely that the strong biofilm-inhibiting properties of JBC 1847 previously documented in *S. aureus*, *Staphylococcus epidermidis*, and *Cutibacterium acnes* (Ronco et al., 2021b) are associated with JBC 1847-blocking of the histidine biosynthesis. In the same way, we have in the present study shown that JBC 1847 is active against bacteria in a slow metabolic state (stationary phase) characteristic of bacterial cell grown in biofilm. In addition to genes involved in the histidine biosynthesis, the two histidine kinase encoding genes, *nra*A and *nre*B, were among the DEGs that were most downregulated by JBC 1847. Recently, histidine kinase inhibitors have been suggested as novel antimicrobial drug targets due to their importance in various processes (Bem et al., 2015).

The finding of only downregulated DEGs in the transcriptome of JBC 1847 treated *S. aureus* indicates a “shut-down” of the bacterial overall viability, which is supported by a significant inhibition in the synthesis rate of all investigated macromolecules (DNA, RNA, cell wall, and protein) (Figure 2).

As JBC 1847 led to complete depolarization of the cytoplasmic membrane (Figure 1), the PMF of the bacteria dissipates. PMF is the driving force for ATP synthesis, and in support of this, we found that JBC 1847 led to a marked decrease in intracellular ATP (Figure 6). Since the membrane potential, PMF, and intracellular ATP level influence pH homeostasis, membrane transport, motility cell division, bacterial communication, and environmental sensing (Benarroch and Asally, 2020), JBC 1847 targets many vital bacterial processes. A correlation between antibiotic-induced decreased intracellular ATP level and increased killing of bacteria has been shown in *Mycobacteria tuberculosis* (Rao et al., 2008), which is in agreement with previous reports on significant antimicrobial effect of phenothiazines on *M. tuberculosis* in particular (Amaral and Molnar, 2010). The downregulation of numerous genes in the histidine metabolism observed in this study could also be a result of limited ATP, as the initial step of the histidine biosynthesis is ATP dependent (Ohta et al., 2000). It is therefore not possible to differentiate if downregulated DEGs of the histidine biosynthesis is a primary cause of JBC 1847 exposure or secondary to the depletion of cellular ATP. Nevertheless, both the inhibition of histidine biosynthesis pathway (Lunardi et al., 2013) and inhibition of the ATP synthase (Liu et al., 2020) have been suggested as attractive targets for development of novel antimicrobials.

Whereas the ATP level was markedly decreased, there was an increase in the ATP/ADP ratio in JBC 1847–treated cells. A similar rise in the ATP/ADP ratio has been observed in *Escherichia coli* exposed to antibiotics, in which it was concluded that (relative) ATP accumulation was not a result of increased respiration, but was due to inhibition of energy-consuming processes (Akhova and Tkachenko, 2014). This is in accordance with the observations on decreased synthesis of macromolecules in our study, processes all depending on energy. In addition, other studies have found that a high ATP/ADP ratio limits the ATP synthesis rate (Meyrat and von Ballmoos, 2019), which could also contribute to a lowered cellular concentration of ATP, in accordance with the results of the present study (Figure 7). The uncoupling of the cellular respiration and, hence, lower level of ATP has been associated with a potentiation of bactericidal antibiotics (Lobritz et al., 2015) which is characteristic for kinetics of JBC 1847. Bedaquiline, an antibiotic used for treatment of tuberculosis, also targets ATP synthase, uncoupling the cellular respiration from ATP synthesis, and as consequence, a futile proton cycle that is linked to cell death is induced (Hards et al., 2015).

In the lack of oxygen, nitrate can serve as anaerobic electron acceptor in the electron chain of *S. aureus* (Burke and Lascelles, 1975); hence, it aids in extrusion of protons from the cellular cytoplasm to generate a PMF (Chen and Strous, 2013). In addition, nitrate reduction can also be coupled to the fermentation of organic acids such as formate to allow for survival in the presence of compounds that dissipate PMF. Therefore, it was somewhat surprising to find that genes essential for nitrate reductase were among the most downregulated. In *S. aureus* exposed to nisin, another membrane depolarization compound, genes encoding nitrate and nitrate reductase were up to 12 times upregulated (Muthaiyan et al., 2008), most likely to compensate for the diminished PMF. In contrast, our results indicate that JBC 1847 inhibits the use of nitrate as an alternative electron acceptor, hence, is not able to counteract the diminished PMF by this pathway.

For the spontaneous mutation assay, we were not able to detect a single *S. aureus* or *E. faecium* isolate with increased tolerance to JBC 1847, in contrast to the relatively high resistance frequencies of the same species against fusidic acid and, to a lesser extent, mupirocin (Table 1). The finding of these low spontaneous resistance frequencies of JBC 1847 is somewhat surprising, as bacterial resistance to uncouplers have been described numerous times for both Gram-positive and Gram-negative bacteria (Krulwich et al., 1990; Lewis et al., 1994; Čubopová et al., 2004). However, the multitarget activity of JBC 1847 as proposed by the results in the present study (uncoupling effect combined with inhibition of the histidine biosynthesis and nitrate reductase pathways) seems difficult for the bacteria to counteract with an increased tolerance response, as shown in the present study for single-step mutations and in a previous study using an induced tolerance (daily sub-culturing, low concentrations) assay (Ronco et al., 2020).

## Conclusion

Multidrug-resistant pathogens infecting humans and animals constitute a serious One Health issue worldwide and, therefore, there is a need for novel antimicrobial treatment solutions. Here, we have investigated the effect of JBC 1847, a potential new antimicrobial compound, on *S. aureus*. We have shown that JBC 1847 causes depolarization of the cell membrane resulting in dissipation of the proton motive (PMF), whereby many essential bacterial processes are affected. Concordantly, JBC 1847 caused a lowered intracellular level of ATP, decreased the synthesis of macromolecules (DNA, RNA, cell wall, and protein) and mediated downregulation of genes essential for the amino acid metabolism. Since JBC 1847 seems to target essential, non-redundant bacterial mechanisms, compensatory mechanisms seem to be limited supported by the observed low rate of resistance toward the compounds. Future studies are needed to verify that the bacterial impact of JBC 1847 suggested in the present study is corresponding to the activity of the compound in *in vivo* settings, and further addressing the degree of JBC 1847 eukaryotic cytotoxicity.

## Data Availability Statement

The datasets presented in this study can be found in online repositories. The names of the repository/repositories and accession number(s) can be found below: https://www.ncbi.nlm.nih.gov/genbank/, NC_001422.1.

## Author Contributions

AS, MM, VA, LK, NB, SS, LS, MA, and AF: experimental work. TR, LS, and KH: statistics, figures, and tables. TR, LK, JC, and RO: validation of results. TR and RO: wrote the manuscript. TR, AP, RO, KH, AP, and JK: manuscript review. JC, RO, AS, and BJ: experimental design. All authors contributed to the article and approved the submitted version.

## Conflict of Interest

AP was employed by company Unibrains. LS and KH were employed by company Bioneer A/S. The remaining authors declare that the research was conducted in the absence of any commercial or financial relationships that could be construed as a potential conflict of interest.

## Publisher’s Note

All claims expressed in this article are solely those of the authors and do not necessarily represent those of their affiliated organizations, or those of the publisher, the editors and the reviewers. Any product that may be evaluated in this article, or claim that may be made by its manufacturer, is not guaranteed or endorsed by the publisher.

## Supplementary Material

Supplementary Figure 1 Molecular structure of JBC 1847 **(A)**, T5 **(B)**, and promazine **(C)**.

Supplementary Figure 2 CFU/ml of *Staphylococcus aureus* JE2 exposed to 1/4 MIC of either JBC 1847 (0.25 mg/L), T5 (0.5 mg/L), promazine (8 mg/L), or left untreated.

## References

[B1] Akhova A. V. Tkachenko A. G. (2014). ATP/ADP alteration as a sign of the oxidative stress development in *Escherichia coli* cells under antibiotic treatment. *FEMS Microbiol. Lett.* 353 69–76. 10.1111/1574-6968.12405 24612220

[B2] Amaral L. Molnar J. (2010). Therapy of XDR TB with thioridazine a drug beyond patent protection but eligible for patent “As New Use”. *Recent Pat. Antiinfect. Drug Discov.* 5 109–114. 10.2174/157489110791233540 20156179

[B3] Anderl J. N. Zahller J. Roe F. Stewart P. S. (2003). Role of nutrient limitation and stationary-phase existence in *Klebsiella pneumoniae* biofilm resistance to ampicillin and ciprofloxacin. *Antimicrob. Agents Chemother.* 47 1251–1256. 10.1128/AAC.47.4.1251-1256.2003 12654654 PMC152508

[B4] Bem A. E. Velikova N. Pellicer M. T. Baarlen P. Van Marina A. Wells J. M. (2015). Bacterial histidine kinases as novel antibacterial drug targets. *ACS Chem. Biol.* 10 213–224. 10.1021/cb5007135 25436989

[B5] Benarroch J. M. Asally M. (2020). The microbiologist’s guide to membrane potential dynamics. *Trends Microbiol.* 28 304–314. 10.1016/j.tim.2019.12.008 31952908

[B6] Burke K. A. Lascelles J. (1975). Nitrate reductase system in *Staphylococcus aureus* wild type and mutants. *J. Bacteriol.* 123 308–316. 10.1128/jb.123.1.308-316.1975 1141199 PMC235721

[B7] Butler M. M. Skow D. J. Stephenson R. O. Lyden P. T. LaMarr W. A. Foster K. A. (2002). Low frequencies of resistance among *Staphylococcus* and *Enterococcus* species to the bactericidal DNA polymerase inhibitor N3-hydroxybutyl 6-(3′-ethyl-4′-methylanilino) uracil. *Antimicrob. Agents Chemother.* 46 3770–3775. 10.1128/AAC.46.12.3770-3775.2002 12435675 PMC132772

[B8] Cassini A. Högberg L. D. Plachouras D. Quattrocchi A. Hoxha A. Simonsen G. S. (2019). Attributable deaths and disability-adjusted life-years caused by infections with antibiotic-resistant bacteria in the EU and the European Economic Area in 2015: a population-level modelling analysis. *Lancet Infect. Dis.* 19 56–66. 10.1016/S1473-3099(18)30605-4 30409683 PMC6300481

[B9] Chen J. Strous M. (2013). Denitrification and aerobic respiration, hybrid electron transport chains and co-evolution. *Biochim. Biophys. Acta Bioenerg.* 1827 136–144. 10.1016/j.bbabio.2012.10.002 23044391

[B10] Čubopová L. Šurín S. Majerník A. Šmigáň P. (2004). Isolation and characterization of an uncoupler-resistant mutant of *Methanothermobacter thermautotrophicus*. *FEMS Microbiol. Lett.* 233 23–28. 10.1016/j.femsle.2004.01.033 15043865

[B11] Fey P. D. Endres J. L. Yajjala V. K. Yajjala K. Widhelm T. J. Boissy R. J. (2013). A genetic resource for rapid and comprehensive phenotype screening of nonessential *Staphylococcus aureus* genes. *mBio* 4:e0537-12. 10.1128/mBio.00537-12.Editor PMC3573662 23404398

[B12] Grein F. Müller A. Scherer K. M. Liu X. Ludwig K. C. Klöckner A. (2020). Ca^2+^-Daptomycin targets cell wall biosynthesis by forming a tripartite complex with undecaprenyl-coupled intermediates and membrane lipids. *Nat. Commun.* 11:1455. 10.1038/s41467-020-15257-1 32193379 PMC7081307

[B13] Hadji-Nejad S. Rahbar M. Mehrgan H. (2010). Synergy between phenothiazines and oxacillin against clinical isolates of methicillin-resistant *Staphylococcus aureus*. *Trop. J. Pharm. Res.* 9 243–249. 10.4314/tjpr.v9i3.56284

[B14] Hards K. Robson J. R. Berney M. Shaw L. Bald D. Koul A. (2015). Bactericidal mode of action of bedaquiline. *J. Antimicrob. Chemother.* 70 2028–2037. 10.1093/jac/dkv054 25754998

[B15] Jana B. Baker K. R. Guardabassi L. (2017). Macromolecule biosynthesis assay and fluorescence mode(s) of action. *Antimicrob. Pept. Methods Protoc.* 1548 181–190. 10.1007/978-1-4939-6737-7_12 28013504

[B16] Jørgensen N. S. (2020). *An Evaluation of the Antimicrobial Activity of a Novel Synthetic Compound.* Master thesis. Copenhagen: University of Copenhagen.

[B17] Jørgensen N. S. Saaby L. Andersson A. M. Kromann S. Sheikhsamani E. Permin A. (2020). A novel derivative of thioridazine shows low toxicity and efficient activity against gram-positive pathogens. *Antibiotics* 9:327. 10.3390/antibiotics9060327 32549350 PMC7344759

[B18] Kanehisa M. (2019). Toward understanding the origin and evolution of cellular organisms. *Protein Sci.* 28 1947–1951. 10.1002/pro.3715 31441146 PMC6798127

[B19] Kanehisa M. Furumichi M. Sato Y. Ishiguro-Watanabe M. Tanabe M. (2021). KEGG: integrating viruses and cellular organisms. *Nucleic Acids Res.* 49 D545–D551. 10.1093/nar/gkaa970 33125081 PMC7779016

[B20] Kanehisa M. Goto S. (2000). KEGG: kyoto encyclopedia of genes and genomes. *Nucleic Acids Res.* 28 27–30. 10592173 10.1093/nar/28.1.27 PMC102409

[B21] Klitgaard J. K. Skov M. N. Kallipolitis B. H. Kolmos H. J. (2008). Reversal of methicillin resistance in *Staphylococcus aureus* by thioridazine. *J. Antimicrob. Chemother.* 62 1215–1221. 10.1093/jac/dkn417 18836185

[B22] Kmietowicz Z. (2017). Few novel antibiotics in the pipeline, WHO warns. *BMJ* 358:j4339. 10.1136/bmj.j4339 28928155

[B23] Kristiansen J. E. Hendricks O. Delvin T. Butterworth T. S. Aagaard L. Christensen J. B. (2007). Reversal of resistance in microorganisms by help of non-antibiotics. *J. Antimicrob. Chemother.* 59 1271–1279. 10.1093/jac/dkm071 17403708

[B24] Krulwich T. A. Quirk P. G. Guffanti A. A. (1990). Uncoupler-resistant mutants of bacteria. *Microbiol. Rev.* 54 52–65. 10.1128/mmbr.54.1.52-65.1990 2181259 PMC372758

[B25] Langmead B. Salzberg S. L. (2012). Fast gapped-read alignment with Bowtie 2. *Nat. Methods* 9 357–359. 10.1038/nmeth.1923 22388286 PMC3322381

[B26] Lewis K. Naroditskaya V. Ferrante A. Fokina I. (1994). Bacterial resistance to uncouplers. *J. Bioenerg. Biomembr.* 26 639–646. 10.1007/BF00831539 7721726

[B27] Li H. Handsaker B. Wysoker A. Fennell T. Ruan J. Homer N. (2009). The sequence alignment/map format and SAMtools. *Bioinformatics* 25 2078–2079. 10.1093/bioinformatics/btp352 19505943 PMC2723002

[B28] Liu L. Beck C. Nøhr-Meldgaard K. Peschel A. Kretschmer D. Ingmer H. (2020). Inhibition of the ATP synthase sensitizes *Staphylococcus aureus* towards human antimicrobial peptides. *Sci. Rep.* 10:11391. 10.1038/s41598-020-68146-4 32647350 PMC7347559

[B29] Lobritz M. A. Belenky P. Porter C. B. M. Gutierrez A. Yang J. H. Schwarz E. G. (2015). Antibiotic efficacy is linked to bacterial cellular respiration. *Proc. Natl. Acad. Sci. U.S.A.* 112 8173–8180. 10.1073/pnas.1509743112 26100898 PMC4500273

[B30] Lunardi J. Nunes J. E. S. Bizarro C. V. Basso L. A. Santos D. S. Machado P. (2013). Targeting the Histidine pathway in *Mycobacterium tuberculosis*. *Curr. Top. Med. Chem.* 13 2866–2884. 10.2174/15680266113136660203 24111909

[B31] Marcel M. (2013). Cutadapt removes adapter sequences from high-throughput sequencing reads. *EMBnet J.* 17 10–12. 10.1089/cmb.2017.0096 28715235

[B32] Meyrat A. von Ballmoos C. (2019). ATP synthesis at physiological nucleotide concentrations. *Sci. Rep.* 9:3070. 10.1038/s41598-019-38564-0 30816129 PMC6395684

[B33] Miller W. R. Bayer A. S. Arias C. A. (2016). Mechanism of action and resistance to daptomycin in *Staphylococcus aureus* and *Enterococci*. *Cold Spring Harb. Perspect. Med.* 6:a026997. 10.1101/cshperspect.a026997 27580748 PMC5088507

[B34] Moriya Y. Itoh M. Okuda S. Yoshizawa A. C. Kanehisa M. (2007). KAAS: an automatic genome annotation and pathway reconstruction server. *Nucleic Acids Res.* 35 182–185. 10.1093/nar/gkm321 17526522 PMC1933193

[B35] Muthaiyan A. Silverman J. A. Jayaswal R. K. Wilkinson B. J. (2008). Transcriptional profiling reveals that daptomycin induces the *Staphylococcus aureus* cell wall stress stimulon and genes responsive to membrane depolarization. *Antimicrob. Agents Chemother.* 52 980–990. 10.1128/AAC.01121-07 18086846 PMC2258546

[B36] Nehme H. Saulnier P. Ramadan A. A. Cassisa V. Guillet C. Eveillard M. (2018). Antibacterial activity of antipsychotic agents, their association with lipid nanocapsules and its impact on the properties of the nanocarriers and on antibacterial activity. *PLoS One* 13:e0189950. 10.1371/journal.pone.0189950 29298353 PMC5752010

[B37] Ohta D. Fujimori K. Mizutani M. Nakayama Y. Kunpaisal-Hashimoto R. Munzer S. (2000). Molecular cloning and characterization of ATP-phosphoribosyl transferase from *Arabidopsis*, a key enzyme in the histidine biosynthetic pathway. *Plant Physiol.* 122 907–914. 10.1104/pp.122.3.907 10712555 PMC58927

[B38] Price L. B. Stegger M. Hasman H. Aziz M. Larsen J. Andersen S. (2012). *Staphylococcus aureus* CC398: host adaptation and emergence of methicillin resistance in livestock. *mBio* 3:e0305-11. 10.1128/mBio.00305-11 22354957 PMC3280451

[B39] R Core Team (2021). *R: A Language and Environment for Statistical Computing.* Vienna: R Foundation for Statistical Computing.

[B40] Rao S. P. S. Alonso S. Rand L. Dick T. Pethe K. (2008). The protonmotive force is required for maintaining ATP homeostasis and viability of hypoxic, nonreplicating *Mycobacterium tuberculosis*. *Proc. Natl. Acad. Sci. U.S.A.* 105 11945–11950. 10.1073/pnas.0711697105 18697942 PMC2575262

[B41] Ronco T. Aragao F. M. Saaby L. Christensen J. B. Permin A. Williams A. R. (2021a). A new phenothiazine derivate is active against *Clostridioides difficile* and shows low cytotoxicity. *PLoS One* 16:e0258207. 10.1371/journal.pone.0258207 34597343 PMC8486139

[B42] Ronco T. Aragao M. F. Svenningsen S. Christensen J. B. Permin A. Saaby L. (2021b). Efficacy of a novel antimicrobial hydrogel for eradication of *Staphylococcus epidermidis*, *Staphylococcus aureus* and *Cutibacterium acnes* from preformed biofilm and treatment performance in an in vivo MRSA wound model. *JAC Antimicrob. Resist.* 3:dlab108. 10.1093/jacamr/dlab108 34337409 PMC8320874

[B43] Ronco T. Jørgensen N. S. Holmer I. Kromann S. Sheikhsamani E. Permin A. (2020). A novel promazine derivative shows high *in vitro* and *in vivo* antimicrobial activity against *Staphylococcus aureus*. *Front. Microbiol.* 11:560798. 10.3389/fmicb.2020.560798 33101232 PMC7555839

[B44] Sabat A. J. Tinelli M. Grundmann H. Akkerboom V. Monaco M. Del Grosso M. (2018). Daptomycin resistant *staphylococcus aureus* clinical strain with novel non-synonymous mutations in the *mprF* and *vraS* genes: a new insight into daptomycin resistance. *Front. Microbiol.* 9:2705. 10.3389/fmicb.2018.02705 30459746 PMC6232378

[B45] Seemann T. (2014). Prokka: rapid prokaryotic genome annotation. *Bioinformatics* 30 2068–2069. 10.1093/bioinformatics/btu153 24642063

[B46] Sharma D. Misba L. Khan A. U. (2019). Antibiotics versus biofilm: an emerging battleground in microbial communities. *Antimicrob. Resist. Infect. Control* 8:76. 10.1186/s13756-019-0533-3 31131107 PMC6524306

[B47] Singer A. C. Kirchhelle C. Roberts A. P. (2019). Reinventing the antimicrobial pipeline in response to the global crisis of antimicrobial-resistant infections [version 1; referees: 2 approved]. *F1000Research* 8:238. 10.12688/f1000research.18302.1 30906539 PMC6426076

[B48] Stenger M. Behr-Rasmussen C. Klein K. Grønnemose R. B. Andersen T. E. Klitgaard J. K. (2017). Systemic thioridazine in combination with dicloxacillin against early aortic graft infections caused by *Staphylococcus aureus* in a porcine model: *in vivo* results do not reproduce the *in vitro* synergistic activity. *PLoS One* 12:e0173362. 10.1371/journal.pone.0173362 28278183 PMC5344393

[B49] Wassmann C. S. Lund L. C. Thorsing M. Lauritzen S. P. Kolmos H. J. Kallipolitis B. H. (2018). Molecular mechanisms of thioridazine resistance in *Staphylococcus aureus*. *PLoS One* 13:e0201767. 10.1371/journal.pone.0201767 30089175 PMC6082566

[B50] Zhou Y. H. Xu C. G. Yang Y. B. Xing X. X. Liu X. Qu Q. W. (2018). Histidine metabolism and IGPD play a key role in cefquinome inhibiting biofilm formation of *Staphylococcus xylosus*. *Front. Microbiol.* 9:665. 10.3389/fmicb.2018.00665 29675012 PMC5896262

