# Supplementary figures and images for: Insight Into the Anti-staphylococcal Activity of JBC 1847 at Sub-Inhibitory Concentration
Source: Front Microbiol. 2022 Jan 5;12:786173. doi: 10.3389/fmicb.2021.786173 (PMC8766816; doi:10.3389/fmicb.2021.786173)

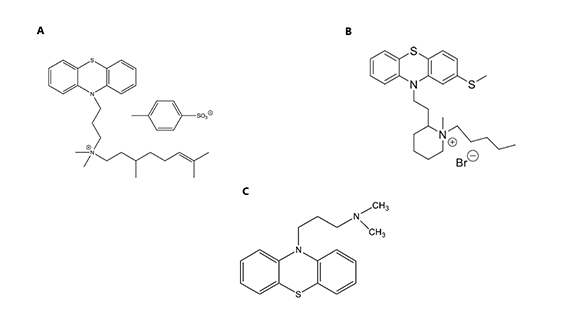

Supplement: Supplementary Figure 1 — Molecular structure of JBC 1847 (A), T5 (B), and promazine (C). [file Image_1.TIF]

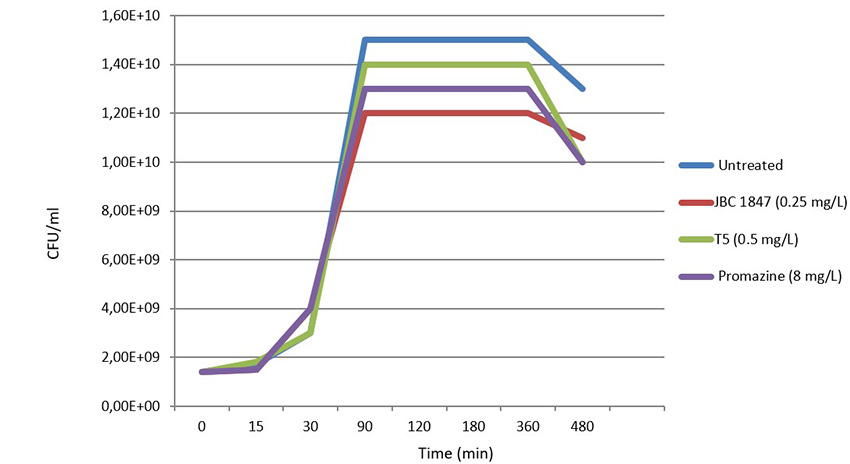

Supplement: Supplementary Figure 2 — CFU/ml of Staphylococcus aureus JE2 exposed to 1/4 MIC of either JBC 1847 (0.25 mg/L), T5 (0.5 mg/L), promazine (8 mg/L), or left untreated. [file Image_2.JPEG]
